# Role of PD-L1 in mediating the effect of lipid on ulcerative colitis: a mediation Mendelian randomization study

**DOI:** 10.3389/fgene.2025.1390605

**Published:** 2025-02-17

**Authors:** Peihong Li, Yiwen Wang, Hongyi Hu, Boyun Sun

**Affiliations:** ^1^ Department of Gastroenterology, Longhua Hospital, Shanghai University of Traditional Chinese Medicine, Shanghai, China; ^2^ Department of Internal Medicine, Tianshan Hospital of Traditional Chinese Medicine, Shanghai, China

**Keywords:** ulcerative colitis, lipids, inflammatory factors, mendelian randomization, mediation analysis

## Abstract

**Introduction:**

Recent evidence suggests that lipids play a crucial role in intestinal metabolic balance and are closely linked to ulcerative colitis (UC). However, the mechanisms underlying their effects remain unclear. This study employed Mendelian randomization (MR) to investigate the relationships among lipids, inflammatory factors, and UC.

**Methods:**

We analyzed data on 179 lipids from the GeneRISK cohort (7,174 individuals), 91 inflammation-related proteins from the EBI GWAS Catalog (14,824 participants), and UC GWAS summary statistics from the FinnGen Biobank (411,317 samples). Associations were assessed using inverse variance weighted (IVW) and Bayesian-weighted MR (BWMR) methods. A mediation analysis was conducted to explore the potential role of inflammatory factors in mediating lipid effects on UC.

**Results:**

MR analysis revealed a significant negative association between sterol ester (27:1/20:4) levels and UC (SNPs = 31; IVW: OR = 0.900 [95% CI: 0.851–0.952], p < 0.001; BWMR: OR = 0.906 [95% CI: 0.849–0.967], p = 0.003). Furthermore, sterol ester (27:1/20:4) was negatively correlated with PD-L1 (SNPs = 30; IVW: OR = 0.961 [95% CI: 0.934–0.990], p = 0.008), and PD-L1 was found to be inversely associated with UC (SNPs = 24; IVW: OR = 0.850 [95% CI: 0.724–0.999], p = 0.048). Mediation analysis suggested that sterol esters (27:1/20:4) may indirectly increase UC risk by downregulating PD-L1 expression. However, the MR analysis results suggest that sterol esters (27:1/20:4) act as a protective factor against UC, which contradicts the mediation analysis. This discrepancy highlights the dual role of PD-L1 in UC pathogenesis.

**Discussion:**

PD-L1 may serve as a key mediator in the regulation of UC pathogenesis by sterol esters, but the underlying complex mechanisms require further investigation.

## 1 Introduction

Ulcerative colitis (UC) is a chronic inflammatory disease characterized by persistent inflammation of the mucosa and submucosa of the colon and rectum (Ungaro et al., 2017). Its global prevalence and incidence are increasing annually, posing a serious health risk and economic burden (Mak et al., 2020). In addition, UC significantly increases the risk of colorectal cancer (Bopanna et al., 2017). Although various factors have been implicated, including genetic defects, epithelial barrier disruption, autoimmune responses, and environmental pollution, the exact pathogenesis of UC remains unclear (Engel and Neurath, 2010). Lipid metabolism is crucial for cellular processes such as signal transduction, development, differentiation, and apoptosis. Dysregulated lipid metabolism has been implicated in several diseases, including diabetes, hyperlipidemia, NAFLD, and cancer (Suvitaival et al., 2018; Wang et al., 2019; Gorden et al., 2015; Białek et al., 2020). In UC, the high turnover of mucosal cells requires increased lipid synthesis to maintain epithelial membrane integrity, highlighting the role of lipid metabolism in its pathogenesis (Lei et al., 2021). Unsaturated fatty acids in phospholipid membranes are highly susceptible to reactive oxygen species, producing lipid peroxidation products such as malondialdehyde and 4-hydroxynonenoic acid. These products disrupt cell membrane structure and impair cellular functions (Ayala et al., 2014; Maiorino et al., 2018). Phosphatidylcholine, a vital component of mammalian cell membranes, plays a key role in maintaining intestinal metabolic homeostasis (Howe and McMaster, 2001; Kennelly et al., 2018). Lipidomic studies have revealed significant alterations in lipid profiles in UC, with changes in phosphatidylcholine levels strongly associated with disease progression (Yu et al., 2023). Notably, phosphatidylcholine 34:1 supplementation increased fumarate level in the mouse colon, suggesting its therapeutic potential in UC. Despite these findings, the precise role of the lipid in UC pathogenesis remains unclear.

Recent studies reveal a strong connection between inflammatory bowel disease (IBD) and metabolic disorders, with the Western diet playing a pivotal role. Excessive nutrient intake in such diets activates the innate immune system and disrupts the intestinal microbiota, establishing a metabolic basis for IBD. These insights suggest that dietary interventions and therapies targeting metabolic pathways could significantly enhance patient outcomes (Adolph et al., 2022). The gut microbiota also directly impacts lipid metabolism by synthesizing and transforming lipids to regulate immune signaling and modifying host cell membrane lipid composition to influence signaling pathways. These findings illuminate the intricate relationships among gut microbiota, lipid metabolism, and IBD (Brown et al., 2023). Chronic inflammation in UC is characterized by an imbalance between pro-inflammatory and anti-inflammatory molecules (Das, 2016). Certain lipids, including sphingolipids and phospholipids, regulate cellular processes such as proliferation, migration, apoptosis, differentiation, and cytokine release, influencing inflammatory pathways (Bryan et al., 2016; Sewell et al., 2012). In UC, phosphatidylcholine is metabolized by phospholipase A2 to release esterified polyunsaturated fatty acids (PUFAs), which form active pro-inflammatory and anti-inflammatory mediators. Lipidomic studies have identified significant changes in lipid profiles in UC patients compared to healthy controls, with alterations in phosphatidylethanolamine (PE) levels correlating with mucosal inflammation (Diab et al., 2019a; Diab et al., 2019b). Given its role in apoptosis, PE has been proposed as a marker for TNF-induced inflammation and a target for cell death imaging (Delvaeye et al., 2018). These findings suggest that lipids may influence UC pathogenesis by modulating inflammatory factor levels. Programmed cell death ligand 1 (PD-L1), a member of the B7 superfamily, is a key regulator of immune responses in UC. PD-L1 interacts with programmed cell death-1 (PD-1) to transmit inhibitory signals, suppressing CD4^+^ and CD8^+^ T cell proliferation and mediating immune tolerance, which can facilitate immune evasion (Pinchuk et al., 2008; Wang and Wu, 2020). Recent studies highlight the upregulation of PD-1/PD-L1 in the mucosal lamina propria and inflammatory cells in UC, particularly on mononuclear cells, correlating with inflammation severity (Roosenboom et al., 2021; Cassol et al., 2020). This suggests a protective feedback mechanism by immune cells during inflammation. PD-L1 plays a critical role in innate and adaptive immune responses and intestinal homeostasis (Chulkina et al., 2020). However, its exact contribution to UC development and progression remains unclear. Further studies on PD-L1 signaling in UC are urgently needed to elucidate its role and therapeutic potential.

Assessing causal effects in observational studies is often hindered by environmental confounding and reverse causation. Genome-wide association studies (GWAS) in large cohorts have advanced our understanding of complex genetic factors in disease. Mendelian randomization (MR) addresses some limitations by using genetic variants associated with the exposure of interest as instrumental variables (IVs) to infer causal relationships (Davey Smith and Ebrahim, 2003). Since genetic variants are randomly assigned at conception, they are less affected by environmental confounders. Recent MR approaches have been applied to mediated pathways (Burgess et al., 2017), leveraging genetic variants as proxies for lifetime exposure to reduce biases from measurement errors common in observational studies. Mediation analysis complements this by elucidating etiological mechanisms and identifying intermediate variables as potential intervention targets, especially when direct exposure modification is challenging (Carter et al., 2021). This study utilized the MR framework to evaluate the causal effects of lipids and inflammatory factors on UC risk. Where evidence of a causal effect of inflammatory factors on UC risk was found, MR mediation analysis was further employed to explore how inflammatory factors mediate the effects of lipids.

## 2 Materials and methods

### 2.1 Study design

**FIGURE 1:**
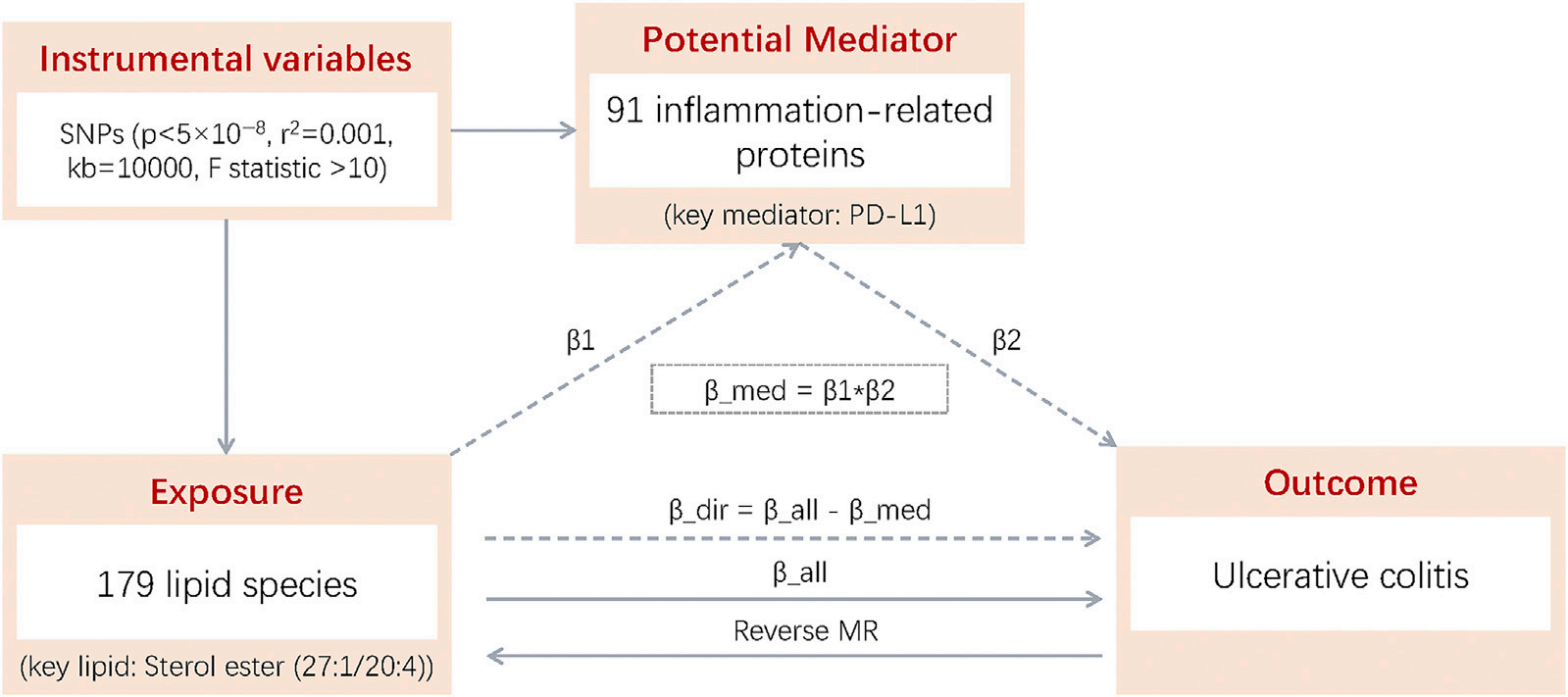
β1: Total effect of 179 lipid species on 91 inflammation-related proteins; β2: Total effect of 91 inflammation-related proteins on UC; β_all; Total effect of 179 lipid species on UC; Reverse MR: MR of UC to 179 lipid species; β_dir: Direct effect of 179 lipid species on UC (β_dir = β_all - β_med); β_med: Mediating effect of 179 lipid species on UC (β_med = β1 × β2).

To evaluate the mediating role of inflammatory factors between lipids and UC, we employed stepwise two-sample MR analysis, using genetic variants as IVs for risk variation. The analysis adhered to three key MR assumptions: (1) a strong association between genetic variation and exposure, (2) no association between genetic variation and confounders, and (3) genetic variation affects the outcome only through the exposure. The stepwise two-sample MR analysis involved three key estimates: (1) the total effect (β_all) of lipids on UC, (2) the direct effect (β1) of lipids on inflammatory factors, and (3) the direct effect (β2) of inflammatory factors on UC. To address potential reverse causality, reverse MR analysis was conducted, treating UC as the exposure to assess its effects on lipids. The calculation of the mediating and direct effects is detailed in Figure 1.

### 2.2 Data sources

The data sources for this study include GWAS datasets on 179 lipids, 91 inflammation-associated proteins, and UC. Data for the 179 lipids were obtained from the GeneRISK cohort, which comprises 7,174 Finnish participants (Ottensmann et al., 2023). This cohort includes blood samples along with ethnographic, health, genotype, and lipidomic data, all managed by the THL Biobank. Summary statistics for 13 lipid classes are publicly available in the GWAS catalog (codes: GCST90277238–GCST90277416). The GWAS data for 91 inflammation-associated proteins were collected from 14,824 individuals, protein quantitative trait locus analyses were conducted for these plasma proteins, measured using the Olink Target platform, to identify genetic influences on inflammatory proteins (Zhao et al., 2023), full summary statistics are publicly available through the Cambridge Epidemiology Unit and the EBI GWAS catalog (codes: GCST90274758–GCST90274848). These 91 proteins were derived from a meta-analysis of 11 cohorts encompassing diverse populations, including individuals with coronary heart disease, neurodegenerative diseases, rheumatoid arthritis, atrial fibrillation, bipolar disorder, as well as blood donors and healthy controls. UC GWAS data were sourced from the FinnGen project, which integrates genetic and health data from 500,000 participants in the Finnish Biobank. The UC cohort included 41,969 European individuals (18,869 females, 22,911 males) with a median age of onset of 37.32 years (females: 36.03 years, males: 38.27 years). The study analyzed 411,317 samples (Ncase = 5,931; Ncontrol = 405,386), encompassing over 520,210 phenotype-related data points. Ethical approval was not required for this study, as it utilized publicly available GWAS summary statistics that had been pre-approved by the relevant ethical review boards. Additional details of the data are presented in Table 1.

**TABLE 1:** Basic information of datasets used in this study.

Trait	Consortium	Ethnicity	Sample size
179 lipid species	Terveyden ja hyvinvoinnin laitos biobank	European	7,174 cases
91 inflammation related proteins	Cardiovascular Epidemiology Unit	European	14,824 cases
Ulcerative colitis	FinnGen	European	411,317 samples (Ncase = 5,931; Ncontrol = 405,386)

### 2.3 Instrumental variables selection

IVs for lipids, inflammatory factors, and UC were screened separately for MR analyses. Exposure-related IVs were identified using a significance threshold of 5 × 10^−8^ and a stringent linkage disequilibrium (LD) criterion (*r* ^2^ = 0.001), excluding SNPs within a 10,000-kb range that did not meet these thresholds (Lawlor et al., 2008; Burgess et al., 2011). To reduce bias from weak instruments, only SNPs with an F-statistic greater than 10 were retained. Palindromic SNPs with mismatched alleles between exposure and outcome were excluded (Cai et al., 2022).

### 2.4 Statistical analyses

Data analysis was conducted using R version 4.3.1 with the ‘Mendelian-Randomization’ package (version 0.4.3). IVW and BWMR were the primary methods used. IVW provided causal estimates while accounting for horizontal pleiotropy, and BWMR validated these results, addressing polygenic effects and pleiotropy with high efficiency and stability. Additional methods, including MR Egger, Weighted Median, Simple Mode, and Weighted Mode, were applied to ensure robustness. Significance thresholds were set at p < 0.05, with Bonferroni adjustments for multiple testing. Heterogeneity was assessed using Cochran’s Q test and funnel plots, while horizontal pleiotropy was evaluated with MR Egger regression (p > 0.05 indicating absence) (Hemani et al., 2018). Sensitivity analysis, including leave-one-out analysis, further validated the robustness of the results. Mediation analysis decomposed total effects into direct and indirect components. The mediation effect was calculated as β1 × β2, with confidence intervals determined via the delta method. The proportion mediated was derived by dividing the indirect effect by the total effect. A p value below 0.05 suggested a statistical significance.

## 3 Results

### 3.1 MR analysis of lipid-causal links to UC

**FIGURE 2:**
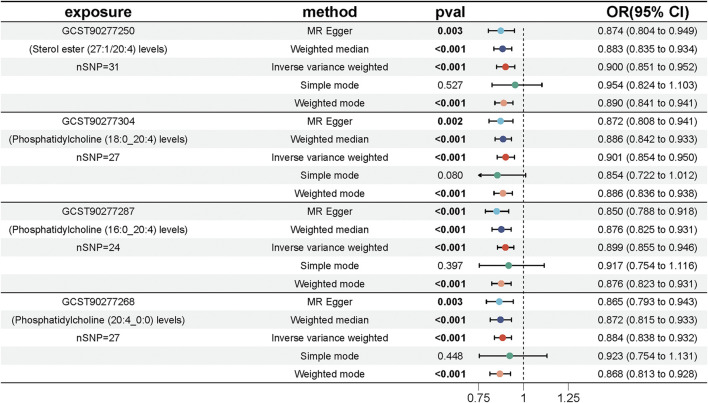
Forest plot showing the effect of four key lipids on the risk of UC. Estimates are shown as ORs and 95% CIs from five methods of MR analysis, including IVW, MR Egger, weighted median, simple model and weighted model.

We performed MR analyses to examine the causal relationships between genetically predicted lipid levels and UC, utilizing 554 SNPs strongly associated with lipids as IVs (Supplementary Table S1). The IVW method with multiplicative random effects was chosen as the primary analytical approach due to the presence of heterogeneity observed in some of the lipid associations. Four lipids met both the Bonferroni-corrected (P < 0.00028) and FDR-corrected (P < 0.05) significance thresholds: Phosphatidylcholine (20:4_0:0) (OR = 0.884, 95% CI = [0.838, 0.932], P = 6.14 × 10^−6^), Phosphatidylcholine (16:0_20:4) (OR = 0.899, 95% CI = [0.855, 0.946], P = 4.31 × 10^−5^), Phosphatidylcholine (18:0_20:4) (OR = 0.901, 95% CI = [0.854, 0.950], P = 1.12 × 10^−4^), and Sterol ester (27:1/20:4) (OR = 0.900, 95% CI = [0.851, 0.952], P = 2.46 × 10^−4^) (Figure 2; Supplementary Table S2).The MR-Egger intercept terms indicated no significant directional pleiotropy for all lipids. However, Cochran’s Q test revealed significant heterogeneity in the association for Sterol ester (27:1/20:4) (Q = 46.41, P = 0.021) (Supplementary Table S3; Supplementary Figure S1), suggesting potential variability in the underlying causal effect. Given the observed heterogeneity in the data, we prioritized the IVW method with multiplicative random effects, which accounts for such variability in the analysis. A Bayesian-weighted validation analysis (OR = 0.906, [95% CI: 0.849–0.967], p = 0.003) yielded similar results to the primary IVW analysis, further confirming the robustness and stability of these causal inferences (Supplementary Table S4).

### 3.2 Assessing potential causal relationships between inflammatory factors and UC

**FIGURE 3:**
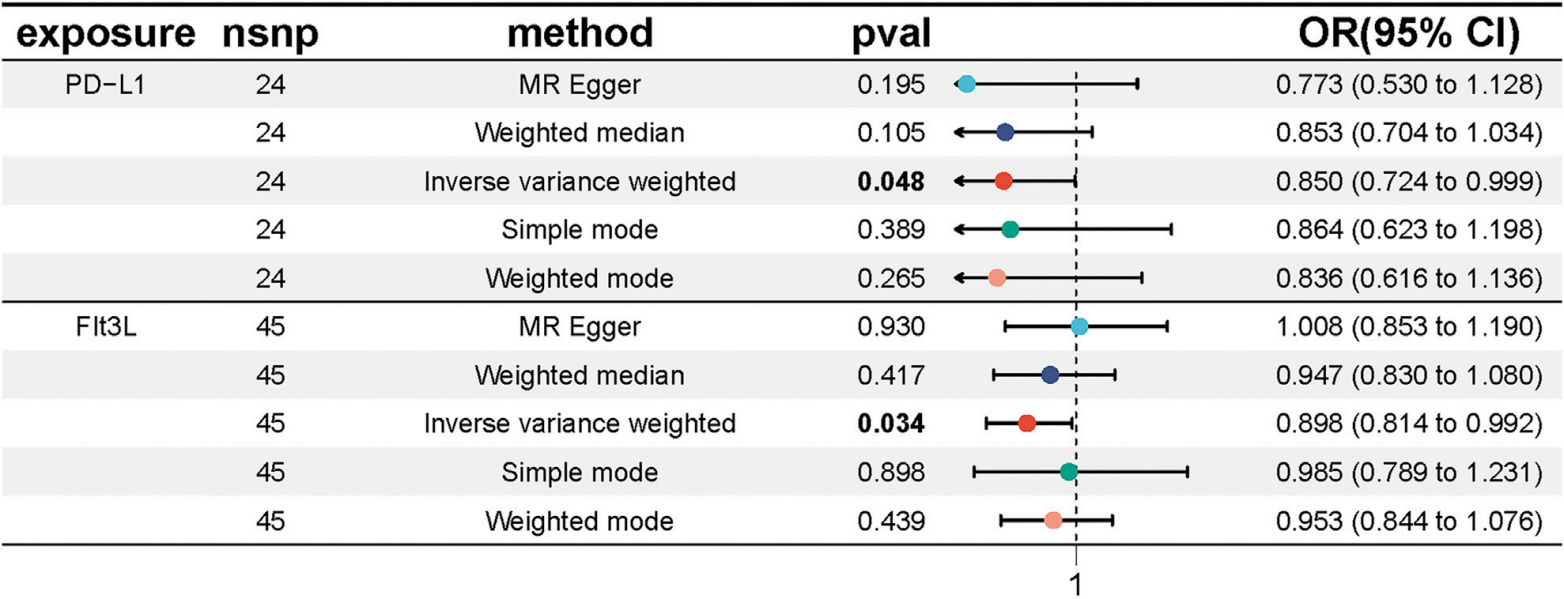
Forest plot showing the causal effect of inflammatory factors on UC risk. The figure shows the OR and 95% CI of the estimates derived from 5 MR analysis methods, including IVW, MR Egger, weighted median, simple model and weighted model.

We conducted MR analyses to assess potential causal relationships between 91 inflammatory factors and UC, identifying two key mediators, FIt3L and PD-L1. IVs for these analyses were selected from 309 SNPs that were strongly associated with inflammatory factors (Supplementary Table S5). The MR-Egger intercept for FIt3L revealed no significant evidence of directional pleiotropy (intercept = −0.015, P = 0.104); however, Cochran’s Q test indicated heterogeneity (Q = 65.66, P = 0.015). Similarly, the MR-Egger intercept for PD-L1 also showed no significant pleiotropy (intercept = 0.009, P = 0.591), but Cochran’s Q test identified heterogeneity (Q = 42.78, P = 0.005) (Supplementary Table S6; Supplementary Figure S2). Given the detected heterogeneity, we used the IVW method under a multiplicative random effects model as the primary analytical approach. The IVW results indicated a potential causal relationship between FIt3L, PD-L1, and UC, with OR of 0.850 (95% CI = [0.724, 0.999], P = 0.048) for FIt3L and 0.898 (95% CI = [0.814, 0.992], P = 0.034) for PD-L1 (Figure 3; Supplementary Table S7).

### 3.3 Causal relationships between lipids and inflammatory factors: insights into sterol ester (27:1/20:4) and PD-L1

**FIGURE 4:**
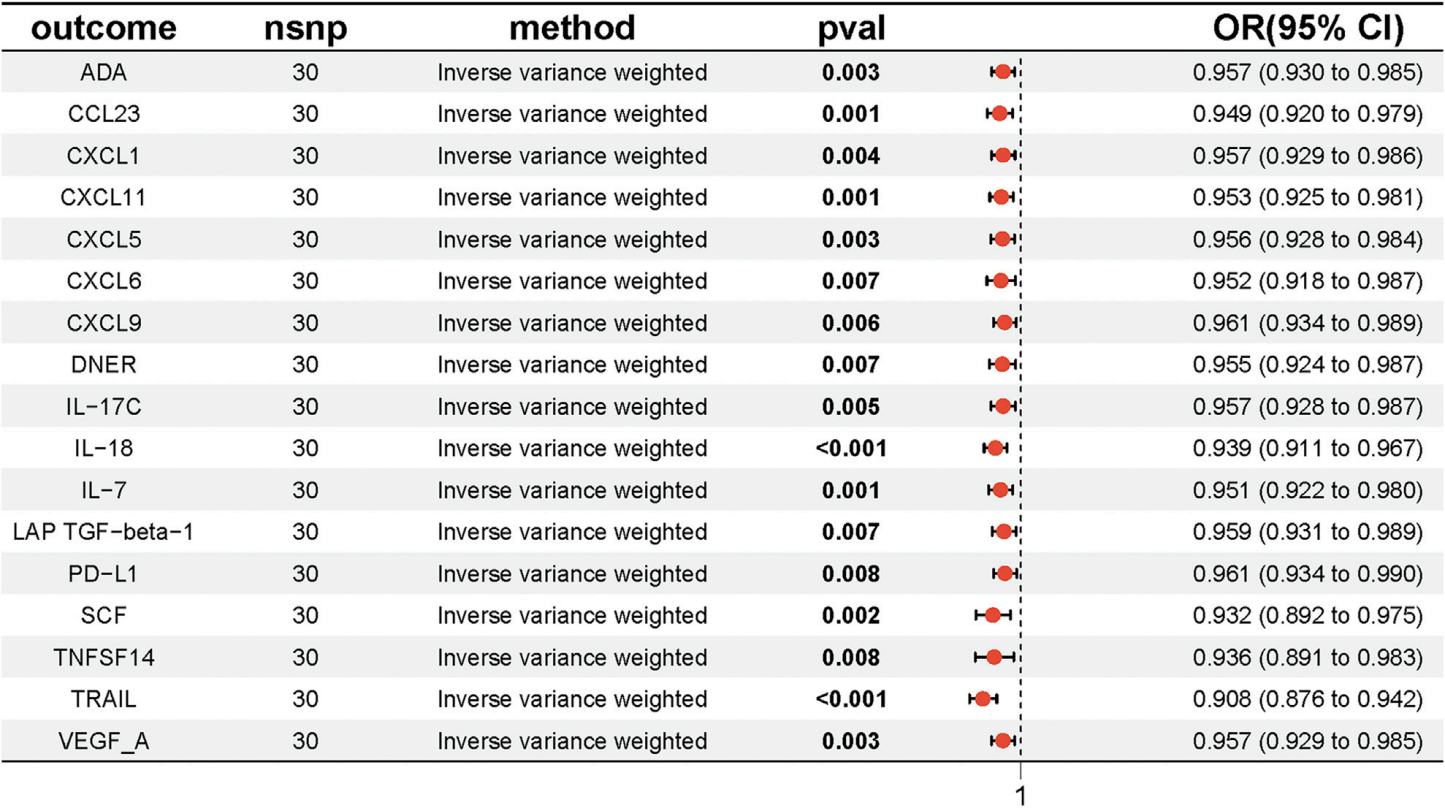
Forest plot showing the effect of Sterol ester (27:1/20:4) on inflammatory factors, with ORs and 95% Cis from the IVW method in the MR analysis.

We conducted MR analyses to investigate the potential causal relationships between four lipid species and 91 inflammatory factors, using the IVW method with multiplicative random effects as the primary analytical approach, with results adjusted by FDR correction. The analysis revealed that Sterol ester (27:1/20:4) levels were potentially causally associated with 17 inflammatory factors, including TRAIL, IL-18, CCL23, CXCL11, IL-7, ADA, CXCL5, SCF, VEGF-A, CXCL1, IL-17C, CXCL6, CXCL9, DNER, LAP TGF-beta-1, PD-L1, and TNFSF14 (P < 0.05). Similarly, Phosphatidylcholine (18:0_20:4) levels were associated with six inflammatory factors (TRAIL, CXCL11, CXCL9, TNFSF14, Beta-NGF, and IL-18; P < 0.05), Phosphatidylcholine (16:0_20:4) levels with four inflammatory factors (TRAIL, CXCL11, CXCL9, and DNER; P < 0.05), and Phosphatidylcholine (20:4_0:0) levels with two inflammatory factors (TRAIL and SCF; P < 0.05) (Figure 4; Supplementary Tables S8, S9). Having established that PD-L1 was causally associated with UC, we hypothesized that PD-L1 may mediate the effect of the sterol ester (27:1/20:4) on UC. Based on these findings, we selected Sterol ester (27:1/20:4) as the key lipid and PD-L1 as the sole mediator. The MR analysis confirmed a significant causal relationship between Sterol ester (27:1/20:4) levels and PD-L1 (OR = 0.961, 95% CI = [0.934, 0.990], P = 0.008). Moreover, the MR-Egger intercept indicated no significant directional pleiotropy (intercept = 0.001, P = 0.882), and Cochran’s Q test demonstrated no heterogeneity (Q = 24.782, P = 0.640) (Supplementary Table S10). These findings suggest that Sterol ester (27:1/20:4) levels may influence inflammatory factors, particularly PD-L1, which could play a critical role in inflammatory regulation. The robustness of these results is supported by the absence of pleiotropy and heterogeneity.

### 3.4 Analysis of reverse causality in UC and sterol ester relationships

**FIGURE 5:**
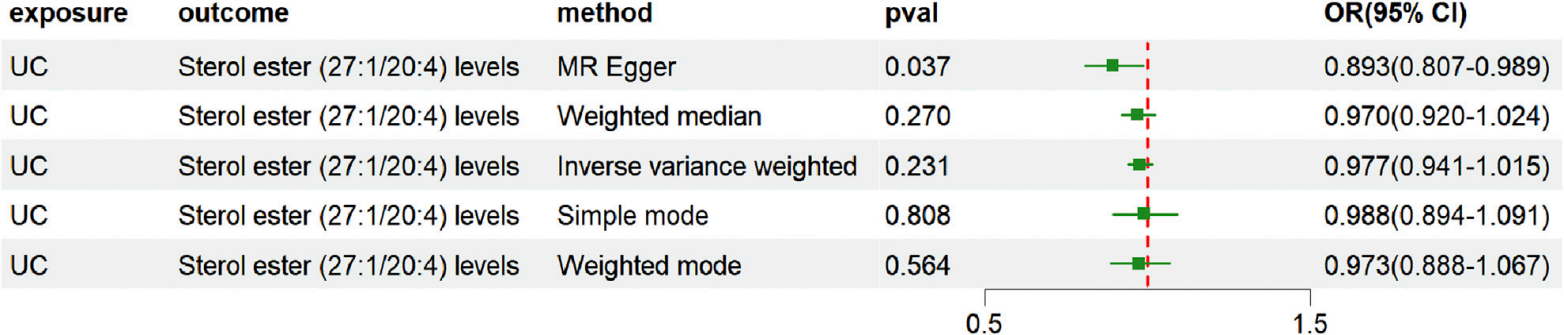
Forest plot showing reverse causality of UC on lipid risk. The figure shows the OR and 95% CI of the estimates derived from 5 MR analysis methods, including IVW, MR Egger, weighted median, simple model and weighted model.

To exclude the possibility of reverse causality between UC and sterol ester levels (27:1/20:4), we conducted an inverse MR analysis. First, the MR-Egger intercept term was examined to assess directional pleiotropy. The results showed no significant evidence of directional pleiotropy (intercept = 0.009, P = 0.348). However, heterogeneity among IVs was detected through Cochran’s Q test (Q = 30, P = 0.02) (Supplementary Table S11). Given the observed heterogeneity, the IVW method under a multiplicative random effects model was selected as the primary analytical approach. The IVW analysis demonstrated no evidence supporting a causal relationship between UC and sterol ester (27:1/20:4) levels, with an OR of 0.977 (95% CI = [0.941, 1.015], P = 0.231) (Figure 5; Supplementary Table S12). These findings indicate that UC does not have a causal impact on sterol ester (27:1/20:4) levels, effectively ruling out the possibility of reverse causality in this association.

### 3.5 Mediating role of PD-L1 in the relationship between sterol ester (27:1/20:4) and UC

This study investigated the potential mediating role of PD-L1, an inflammation-related factor, in the relationship between Sterol ester (27:1/20:4) and UC. The analysis revealed that Sterol ester had a significant negative total effect on UC (β_all = −0.105, 95% CI = [0.851, 0.952]), with a prominent direct inhibitory effect (β_dir = −0.112, 95% CI = [-0.020, 0.033], P = 0.034). The direct effect of Sterol ester on PD-L1 (β1 = −0.039, 95% CI = [0.934, 0.990]) and the direct effect of PD-L1 on UC (β2 = −0.163, 95% CI = [0.934, 0.990]) were both negative, indicating that Sterol ester modulates PD-L1 expression and that PD-L1 negatively influences UC development. In addition, the mediation effect by PD-L1 was positive but relatively small (β_med = 0.006), indicating that PD-L1 slightly attenuated the direct inhibitory effect of sterol ester on UC. This suggests that PD-L1 plays a potential modulatory role in the pathway, likely influencing UC development through its involvement in inflammatory signaling. Although the mediation effect was modest, its biological significance merits further investigation to better understand the complex interactions between sterol ester, PD-L1, and UC, as well as the role of PD-L1 in inflammation-mediated disease processes.

## 4 Discussion

This study employed MR analysis to explore the causal relationship between lipid levels and UC risk. The analysis identified a significant association between elevated levels of sterol esters (27:1/20:4) and a reduced risk of UC. Further mediator MR analysis suggested that PD-L1 may serve as a critical mediator in this relationship. Genetically predicted PD-L1 levels were inversely associated with UC risk, implying that higher levels of sterol esters (27:1/20:4) might suppress PD-L1 expression, thereby increasing susceptibility to UC. These findings underscore the potential role of PD-L1 as a key regulatory element in the UC pathway modulated by sterol esters (27:1/20:4). However, the MR analysis revealed discrepancies between the total effect of sterol esters (27:1/20:4) on UC risk and the mediating effect of PD-L1, suggesting a dual role for PD-L1 in UC pathogenesis. While sterol esters (27:1/20:4) primarily exhibit a protective effect, their interaction with PD-L1 highlights a complex modulatory pathway that warrants further investigation.

Sterol esters (27:1/20:4) are lipid components of plant and animal cell membranes. Plant-derived sterol esters are well-documented for their ability to reduce intestinal cholesterol absorption and lower serum low-density lipoprotein cholesterol levels. Beyond their cholesterol-lowering effects, emerging evidence suggests that sterol esters also play a significant role in modulating immune function. Cellular studies have demonstrated that plant sterol esters reduce the production of pro-inflammatory cytokines and prostaglandins (Awad et al., 2004; Desai et al., 2009). In parallel, animal studies have reported anti-inflammatory effects of phytosterol esters (Navarro et al., 2001; Oliveira et al., 2004), and human intervention trials have shown reductions in inflammatory responses across diverse patient populations (Brüll and Mensink, 2009; Bouic, 2002). These findings collectively underscore the protective role of phytosterol esters, aligning with the results of total effect analyses in MR studies. However, contradictory findings have been reported regarding sterol esters (27:1/20:4) and their potential role in inflammatory pathways relevant to UC. Some studies suggest that sterol esters may indirectly influence inflammatory mediators implicated in UC, such as cytokines, interleukins, nitric oxide, free radicals, Toll-like receptor (TLR) activation, oxyphospholipids, and gut microbiota dysbiosis. These factors, in turn, may elevate UC risk (Das, 2016). This observation is consistent with our mediator MR analyses, which hypothesized that lipids, including sterol esters (27:1/20:4), could contribute to UC pathogenesis by modulating inflammatory mediators such as PD-L1. Our MR analyses revealed a negative correlation between sterol esters (27:1/20:4) and 17 inflammatory factors, reinforcing the hypothesis that sterol esters exert an anti-inflammatory effect. Among these factors, PD-L1 appears to be a key mediator. Notably, increased PD-L1 expression, both at the mRNA and protein levels, has been reported in patients with UC (Mezache et al., 2017; Rajabian et al., 2019; Beswick et al., 2018). Furthermore, clinical observations indicate that 2%–5% of patients treated with anti-PD-1/PD-L1 therapies develop gastrointestinal adverse effects, including structural changes, ulceration, and UC-like lesions (Han et al., 2020; Dougan et al., 2021). Experimental studies in mice have demonstrated that disruption of the PD-1/PD-L1 signaling pathway compromises intestinal mucosal tolerance to autoantigens, resulting in severe autoimmune enteritis (Chulkina et al., 2020). These findings highlight the complex and potentially dual role of PD-L1 in UC pathogenesis. While PD-L1 may act as a mediator of the protective effects of sterol esters, its dysregulation could contribute to disease progression. The mechanisms driving PD-L1 dysregulation in UC remain poorly understood, underscoring the need for further research to elucidate the immunological pathways involved and to identify novel therapeutic targets for UC.

Direct studies investigating the impact of sterol ester (27:1/20:4) on PD-L1 expression are currently lacking. However, findings from our MR analysis suggest that supplementation with sterol ester (27:1/20:4) may result in PD-L1 downregulation. Pro-inflammatory mediators, such as IFN-γ, TNF-α, and IL-17A, are closely associated with PD-L1 expression levels (Kryczek et al., 2008; Karakhanova et al., 2010; Ou et al., 2012), and supplementation with plant-derived sterol esters has been shown to significantly reduce TNF-α and IL-1β levels, indicative of decreased inflammation (Desai et al., 2009; Brüll et al., 2016). Although the anti-inflammatory effects of plant sterol esters have been variably reported, their influence on T cell-specific activity is well-documented. Evidence demonstrates that sterol esters shift immune responses toward a Th1-dominant phenotype in both murine models (Calpe-Berdiel et al., 2007; Lee et al., 2007) and humans, particularly in individuals with atopic diseases (Bouic, 2001) and HIV (Breytenbach et al., 2001). *In vitro* studies further reveal that plant sterol esters induce Th1 responses in peripheral blood mononuclear cells from asthma patients (Brüll et al., 2012), likely through TLR2 activation, enhanced IL-2 production, and improved regulatory T cell (Treg) numbers and function (Brüll et al., 2010). Despite limited exploration of the effects of plant sterol esters on intestinal inflammation, existing studies suggest they mitigate T-cell-dependent intestinal inflammation by reducing CD3^+^ T cells and increasing Tregs (Te et al., 2015). Given the central role of T cells in UC pathogenesis (De Souza and Fiocchi, 2016), these findings hold potential significance. Naïve CD4^+^ T cells differentiate into effector subsets, including Th1, Th2, Th17, Th9, Th22, and Tregs, in response to antigen-presenting cell signaling and cytokine cues (Neurath, 2017). Furthermore, B lymphocytes with high PD-L1 expression can transition from plasma to memory cells, modulating Th1/Th17 activity (Khan et al., 2015). Aguirre et al. (2020) demonstrated that normal fibroblasts inhibit Th1/Th17 activity through the PD-1/PD-L1 pathway. Notably, PD-1 expression on T helper cells positively correlates with disease activity in active UC patients (Long et al., 2021). Dysregulated inflammatory responses, coupled with inadequate regulatory control, are thought to drive chronic enteritis (Ahluwalia et al., 2018), and aberrant PD-1/PD-L1 signaling may underlie the dysregulation of T cell responses in UC. Based on these observations, we hypothesize that sterol ester (27:1/20:4) may modulate immune responses through effects on PD-1/PD-L1 signaling in both mucosal and systemic immune cells. Further research is essential to elucidate the mechanisms by which sterol esters influence PD-L1-mediated pathways and to explore their therapeutic potential in UC management.

This study has several notable strengths. By leveraging summary statistics for exposures and outcomes from the largest and most recent GWAS, we ensured robust and reliable findings while avoiding sample overlap. To enhance statistical power and reduce bias, rigorous IV selection criteria were applied, complemented by the exclusion of SNPs associated with potential confounders. Genetic variations analyzed were distributed across multiple chromosomes, which may help minimize the influence of gene-gene interactions. Furthermore, the use of BWMR preserved data integrity while addressing bias, and corrections such as Bonferroni and FDR were employed to enhance the robustness of the two-sample MR analysis. However, there are also limitations to our study. The GWAS data included only individuals of European ancestry, limiting the generalizability of our findings and necessitating further research to assess mediating effects in non-European populations. Moreover, some heterogeneity was observed due to reliance on GWAS data, restricting our ability to investigate nonlinear associations and stratification effects by factors such as age, health status, or sex. This highlights the need for studies with broader perspectives and larger sample sizes to validate the causal relationships. Finally, we analyzed a limited subset of lipids and inflammatory factors, underscoring the importance of future research to identify additional mediators and elucidate the mechanisms underlying these associations.

## 5 Conclusion

This study highlights a protective association between sterol esters (27:1/20:4) and reduced UC risk, with PD-L1 acting as a dual mediator. While PD-L1 may mediate the protective effects of sterol esters, its dysregulation could contribute to disease progression. These findings emphasize the complex role of PD-L1 in UC and the need for further research to better understand its mechanisms and therapeutic potential.

## Data Availability

The datasets presented in this study can be found in online repositories. The names of the repository/repositories and accession number(s) can be found in the article/Supplementary Material.
