# Supplementary material for: Role of PD-L1 in mediating the effect of lipid on ulcerative colitis: a mediation Mendelian randomization study
Source: Front Genet. 2025 Feb 17;16:1390605. doi: 10.3389/fgene.2025.1390605 (PMC11872926; doi:10.3389/fgene.2025.1390605)
Supplement: Supplementary file 1 [file DataSheet2.docx]

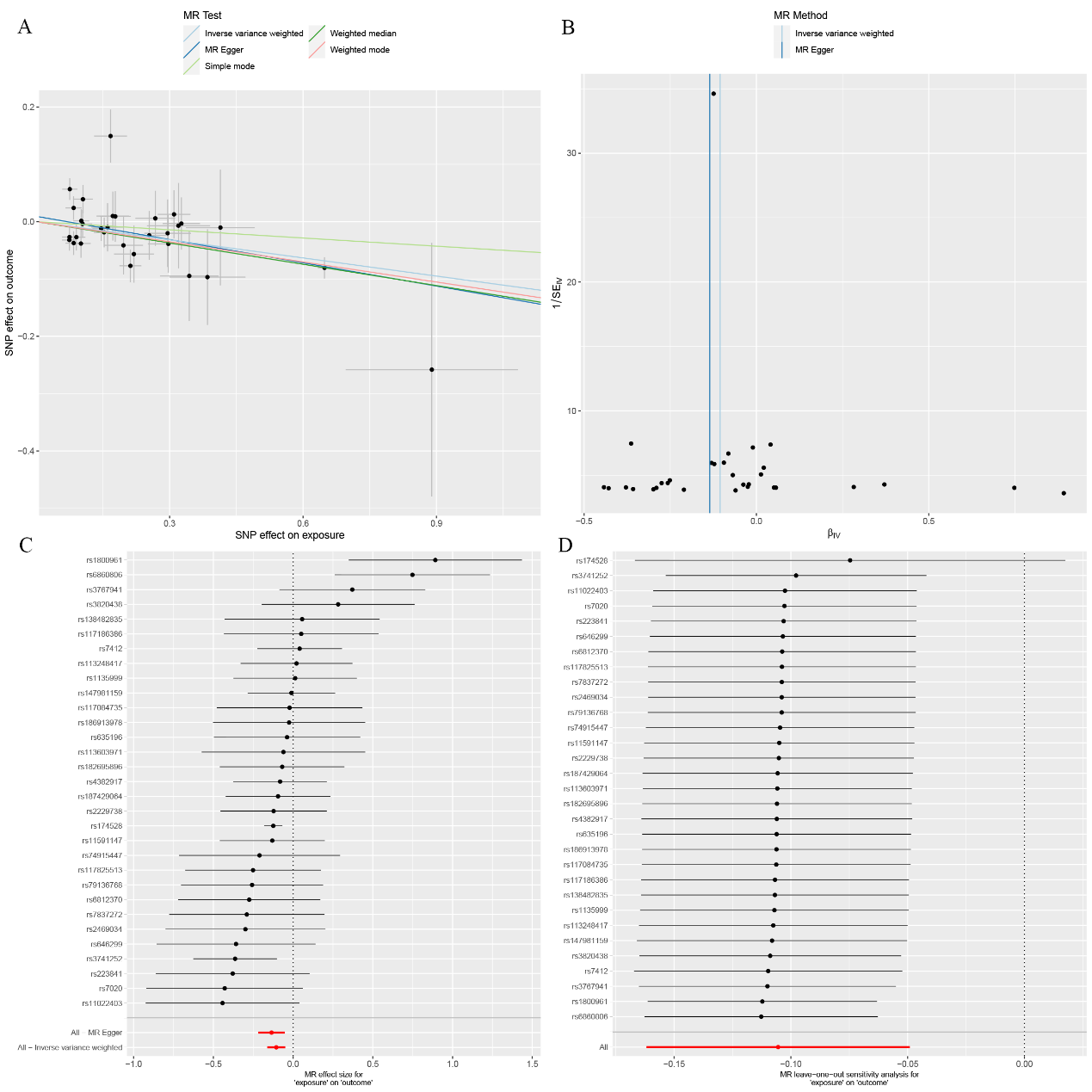


**Figure S1.** (A) Scatter plot illustrating the causal effect of sterol ester (27:1/20:4) levels on ulcerative colitis (UC) risk. The slope of the regression line represents the magnitude of the causal association. (B) Funnel plot assessing the potential bias in the causal relationship between sterol ester (27:1/20:4) levels and UC risk. (C) Forest plot summarizing the results of Mendelian randomization (MR) analyses investigating the causal relationship between sterol ester (27:1/20:4) levels and UC risk. (D) Leave-one-out analysis plot showing the robustness of the causal effect of sterol ester (27:1/20:4) levels on UC risk.


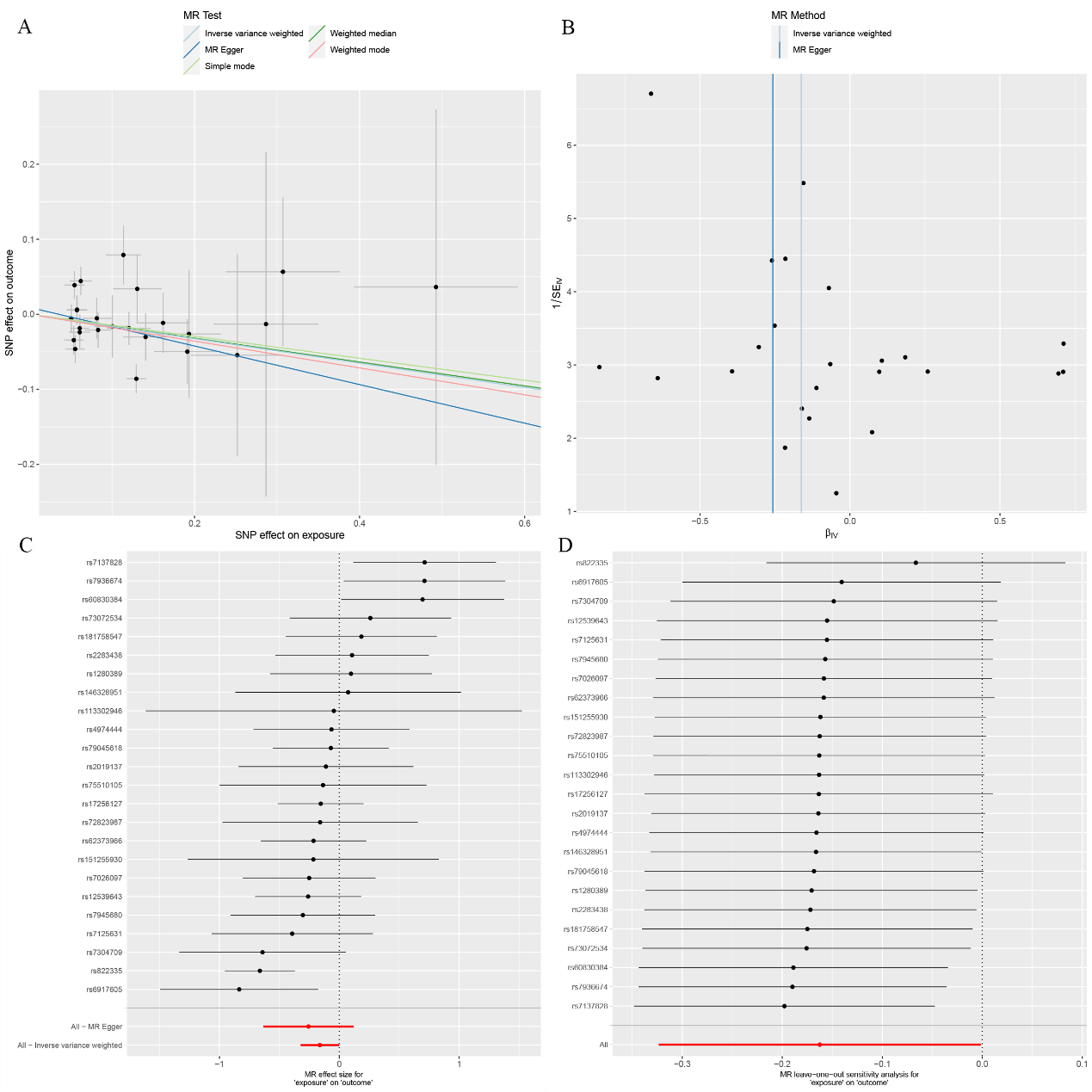


**Figure S2.** (A) Scatter plot illustrating the causal effect of PD-L1 expression on UC risk. The slope of the regression line represents the magnitude of the causal association. (B) Funnel plot assessing the potential bias in the causal relationship between PD-L1 expression and UC risk. (C) Forest plot summarizing the results of MR analyses investigating the causal relationship between PD-L1 expression and UC risk. (D) Leave-one-out analysis plot showing the robustness of the causal effect of PD-L1 expression on UC risk.
